# Real-world data-driven early warning system for risk-stratified liver injury in hospitalized COVID-19 patients—Machine learning models for clinical decision support

**DOI:** 10.3389/fpubh.2025.1566260

**Published:** 2025-09-03

**Authors:** Yuanguo Xiong, Xu Cai, Xin Lai, Yuwen Wang, Hao Xin, Wei Song, Feng Lv, Xianxi Guo, Ge Yang, Yue Wu

**Affiliations:** ^1^Department of Pharmacy, Renmin Hospital of Wuhan University, Wuhan, China; ^2^Department of Pharmacy, The First Affiliated Hospital of Nanchang University, Nanchang, China; ^3^Department of Clinical Pharmacy, People's Hospital of Macheng, Huanggang, China; ^4^Department of Pharmacy, Qingdao Third People's Hospital Affiliated to Qingdao University, Qingdao, China; ^5^Department of Pharmacy, First Affiliated Hospital of Army Medical University, Chongqing, China

**Keywords:** COVID-19, liver injury, machine learning, random forest, extra trees, SHAP analysis

## Abstract

**Objective:**

To develop and validate a real-world evidence-driven early warning system for the risk-stratified prediction of coronavirus disease 2019 (COVID-19)-associated hepatic dysfunction in hospitalized patients, leveraging interpretable machine learning models to provide clinically actionable decision support for timely intervention.

**Methods:**

A retrospective single-center cohort study was conducted utilizing high-resolution electronic health records (EHRs) from 983 hospitalized COVID-19 patients. Clinical features (e.g., laboratory results, medication exposures, and disease progression markers) were systematically analyzed. To mitigate class imbalance, we employed the Synthetic Minority Oversampling TEchnique (SMOTE) prior to model development. Thirteen distinct machine learning (ML) algorithms were trained and benchmarked to construct an optimal risk stratification framework. Model performance was rigorously evaluated using metrics, including accuracy, precision, recall, F1-score, and area under the receiver operating characteristic curve (AUC). SHapley Additive exPlanations (SHAP) analysis was employed to enhance clinical interpretability and provide transparent insights for decision-making.

**Results:**

The SMOTE-edited nearest neighbors (ENN) technique (SMOTE-ENN) resampling strategy, combined with random forest (RF) and extra trees (ET) models, demonstrated superior predictive performance, achieving AUC values of 0.998 ± 0.002 (RF) and 0.997 ± 0.002 (ET), respectively. The SHAP-based interpretability analysis identified glutathione administration and hepatic enzymes (e.g., gamma-glutamyltransferase [GGT] and alanine aminotransferase [ALT]) as the most influential predictors. The online prediction platforms were developed for liver injury early warning risk stratification (low- and high-risk) based on predicted probabilities classification.

**Conclusion:**

This research successfully established a machine learning-powered early warning system capable of real-time risk stratification for COVID-19-associated liver injury through dynamic integration of clinical data. The ensemble RF/ET-based models demonstrated significant clinical utility as decision support tools, particularly through their ability to identify high-risk patients requiring intensified monitoring and optimize hepatoprotective. By emphasizing drug-induced injury markers and disease progression process, ML models establish a personalized monitoring framework that could potentially transform clinical management for target patients.

## 1 Introduction

The coronavirus disease 2019 (COVID-19) pandemic, caused by severe acute respiratory syndrome coronavirus disease 2 (SARS-CoV-2) virus, has profoundly serious impacted on public health and resulted in a significant loss in economy all over the world (1–3). The COVID-19 pandemic continues due to the ongoing mutations of the SARS-CoV-2 virus (4). Earlier studies demonstrated that SARS-CoV-2 infection not only impacts the respiratory system but also affects other organs, leading to multiple organ failure and a subsequent increase in the all-cause mortality rate (5, 6). Emerging evidence suggests that hepatic dysfunction is involved in up to 50% of COVID-19 cases, with liver injury significantly contributing to the elevated mortality rates observed (6–8). Additionally, it may require specific therapeutic interventions (7, 9, 10). Consequently, COVID-19 combined with liver injury has emerged as a significant clinical challenge; vigilant monitoring of liver function is essential among hospitalized COVID-19 patients (61).

Artificial intelligence (AI) is a broad term that encompasses the use of computers to develop intelligent models capable of performing specified functions, with a significant impact on research in the natural and social sciences (11, 12). Due to the availability of large datasets worldwide and the recent advancements, such as object recognition and decision-making systems, there has been a significant increase in AI applications (13, 14). Machine learning (ML) is a series of computer algorithms employed by AI, to generate predictive models, which have been proven more efficient than traditional methods for corporations with large datasets (15). Many studies have applied ML techniques to facilitate disease progression prediction in medicine, making ML-based models valuable for implementation in clinical practice (16). This demonstrates that AI studies showed a meaningful impact on clinical practice and are worth taking seriously due to their potential benefits in the real world.

In aspects of the COVID-19 pandemic, several AI models have been applied to the disease's diagnosis, prognosis, and outcome. Seyed et al. compared a series of ML algorithms and indicated that a random forest (RF) predictive model had the highest accuracy in mortality prediction of COVID-19 patients, which could benefit from appropriate care for the highest risk (17). To predict the clinical outcomes of hospitalized COVID-19 patients treated with remdesivir, Antonio et al. tested six supervised ML methods and found that extreme gradient boost (XGB) achieved the highest accuracy in mortality (95.45%) and hospital stay length (94.24%) (18). Another cohort study, conducted by Ramón et al., also showed that the XGB method achieved the highest accuracy (93.16%) among other ML methods in predicting the outcome of patients associated with severe respiratory failure treated with tocilizumab (19). These tools could help in making an effective and impactful treatment strategy to optimize the management of COVID-19 patients. Nevertheless, there is a paucity of research on hepatic injury, a significant complication among individuals afflicted with COVID-19. Currently, the diagnosis of liver damage continues to rely on conventional diagnostic modalities, and there is an urgent need to develop expeditious and precise predictive methodologies. This area requires further investigation and enhancement.

To address the critical clinical challenge, we developed an ML model-based early warning system designed for risk-stratified liver injury among hospitalized COVID-19 patients, utilizing real-world data. The Synthetic Minority Oversampling TEchnique (SMOTE) was applied to address the dataset imbalance. Subsequently, several ML algorithms were trained and evaluated for their performance. Finally, the SHapley Additive exPlanations (SHAP) algorithm was used to interpret the optimized model. Additionally, we have developed an effective and accurate online tool to help clinicians manage COVID-19 patients with ease.

## 2 Methods

### 2.1 Study population and ethics approval

This single-center, retrospective cohort study included hospitalized COVID-19 patients from December 2022 to June 2023 at the Renmin Hospital of Wuhan University, a leading tertiary hospital with ~7,300 beds in China. This study has received approval from the ethics committee of clinical research (approval number: WDRY2024-K003), and the informed consent requirement was waived.

### 2.2 Patient inclusion and exclusion criteria

An inpatient diagnosed with COVID-19 (the COVID-19 nucleic acid test was positive) receives antiviral and symptomatic treatment as recommended by the guidelines included in this study (20, 21). Individuals were excluded based on the following criteria: (1) COVID-19 nucleic acid test result was negative; (2) insufficient essential laboratory tests; and (3) severe liver injury at admission.

### 2.3 Data collection and preprocessing

Electronic medical records of enrolled patients were meticulously reviewed. Patients' demographic characteristics, duration of hospitalization, severity of COVID-19, comorbidities, medications, liver function tests, routine blood tests, coagulation parameters tests, and infection indicators were collected. To maintain data integrity and accuracy, we implemented a rigorous dual-entry verification process, where all data were independently recorded and cross-validated by two separate researchers. Any duplicate or anomalous values were systematically identified, rigorously reviewed, and appropriately reconciled to ensure data quality (22, 23). Then, all data were systematically extracted into standardized forms. Categorical variables were dichotomized (presence = 1, absence = 0), while continuous variables underwent standardized unit conversion and normalization to maintain dataset consistency (24, 25). Finally, to minimize bias, variables with more than a 35% missing data threshold were excluded. Subsequent missing values were imputed using Multiple Imputation by Chained Equations (MICE), accounting for data uncertainty through chained regression models (26, 27).

### 2.4 Definition of liver injury

Liver injury was defined as biochemical abnormalities in liver function following hospital treatment. The criteria are based on liver function indices exceeding the upper limit of normal (ULN), with the reference thresholds as follows: (1) Alanine aminotransferase (ALT) levels >40 U/L; (2) aspartate transaminase (AST) levels higher than 35 U/L; (3) alkaline phosphatase (ALP) levels higher than 135 U/L; (4) gamma-glutamyltransferase (GGT) levels higher than 45 U/L; and (5) total bilirubin (TBIL) levels higher than 23 μmol/L.

The severity of liver injury was stratified according to biological criteria derived from the ULN for ALT, ALP, and GGT, adhering to the Common Terminology Criteria for Adverse Events (CTCAE) version 5.0 from the National Cancer Institute (NCI). The grading system is as follows: (1) Grade 1 (mild liver injury): ALT levels are elevated to 1 × ULN to ≤ 3 × ULN, or ALP and GGT levels are elevated to ≥1 × ULN and ≤ 2.5 × ULN; (2) grade 2 (moderate liver injury): ALT levels are elevated to ≥3 × ULN and ≤ 5 × ULN, or ALP and GGT levels are elevated to ≥2.5 × ULN and ≤ 5 × ULN; and (3) grades 3 and 4 (severe liver injury): ALT levels are elevated to ≥5 × ULN, or ALP and GGT levels are elevated to ≥5 × ULN.

### 2.5 Definition of the risk-stratified liver injury

Current clinical practice guidelines recommend serial monitoring of hepatic biochemical markers, including ALT, AST, ALP, and TBL, within 48–72 h intervals for COVID-19 patients exhibiting moderate-to-severe liver injury to assess biochemical persistence and disease progression. The high-risk cohort was operationally defined as patients exhibiting moderate-to-severe hepatic dysfunction (ALT > 3 × ULN, AST > 3 × ULN or TBIL > 2 × ULN), whereas the low-risk cohort comprised individuals with either normal hepatic function or only mild impairment (ALT < 3 × ULN, AST < 3 × ULN or TBIL < 2 × ULN). The primary objective of the current research was to develop and validate ML models for the prospective identification of COVID-19 hospitalized patients at elevated risk for progression to moderate or severe liver injury, thereby providing real-time risk stratification for informed clinical decision-making.

### 2.6 Overview of ML models

In our study, we utilized several ML models (28–30), which are detailed as follows: (1) Logistic regression (LR), widely applied in classification tasks, especially for binary classification problems, an effective and simple model owing to its outputs can be interpreted as probabilities; (2) decision trees (DT), one of the predictive modeling techniques used for classification and regression which adopts a tree-like model of decisions and their possible consequences, can handle non-linear relationships without feature scaling and easily visualized; (3) random forest (RF), an ensemble learning method for classification and regression that constructs a multitude of decision trees at training time and outputs the class that is the mode of the individual trees (classification) or the mean prediction of the trees (regression); (4) gradient boosting (GBoost), an ML technique involving a sequence of weak models (like decision trees) in a specific order to minimize a given loss function, which builds the models iteratively; (5) adaptive boosting (AdaBoost), an ensemble meta-algorithm that focuses on misclassified cases, fitting the model iteratively by adjusting the weights of misclassified instances; (6) eXtreme gradient boosting (XGBoost), an optimized distributed gradient boosting library designed to be highly efficient, flexible, and portable, providing a parallel tree boosting algorithm that solves problems quickly and accurately; (7) Naïve bayes (NB), a probabilistic algorithm based on Bayes' theorem, assuming feature independence, is particularly effective for text classification tasks and high-dimensional data; (8) Support Vector Machine (SVM) with RBF kernel, a classification algorithm that finds a hyperplane maximizing margin, mapping data into higher dimensions to handle non-linear relationships through the use of kernel functions; (9) light gradient boosting machine (LightGBM), a gradient boosting framework using tree-based learning algorithms, designed for high efficiency and scalability, handling large datasets and categorical features effectively with a leaf-wise growth strategy; (10) CatBoost, a gradient boosting library optimized for categorical features, employs ordered boosting to prevent overfitting, offering robust performance on tabular data; (11) K-nearest neighbors (KNN) classifier, a simple and instance-based learning algorithm that classifies a data point based on the majority vote of its neighbors, where the distance metric significantly impacts performance and requires normalization for consistent results; (12) extra trees (ET), an ensemble method creating multiple decision trees by randomizing both feature splits and dataset sampling, reducing variance and improving model robustness; and (13) voting classifier (VC), an ensemble approach combining predictions from multiple classifiers, outputting the majority vote (classification) or average (regression) for improved accuracy.

### 2.7 Model evaluation

To address the challenge of dataset imbalance, SMOTE was utilized (31, 32). This method is specifically tailored to balance class distributions, ensuring equitable representation of both classes during model training. A 5-fold cross-validation (5-CV) approach, combined with an independent validation, was implemented to minimize the risk of overfitting (33, 34). Final performance metrics were computed as the average results across all 5-folds.

The model's performance was optimized using accuracy, precision, recall, and F1-score as key metrics. The calculations for these metrics are outlined in the following formulas:


$$\begin{array}{r} \text{Accuracy =}\frac{\text{True Positives + True Negatives}}{\text{Total number of instances}}\\ \text{Precision =}\frac{\text{True Positives}}{\text{True Positives + False Positives}}\\ \text{Recall = }\frac{\text{True Positives}}{\text{True Positives + False Negatives}}\\ \text{F1-Score = 2}\times \frac{\text{Precision}\times \text{Recall}}{\text{Precision + Recall}} \end{array}$$

Additionally, an area under the curve (AUC) analysis was conducted to evaluate and compare the predictive performance of different models.

### 2.8 Model explainability

To explain the model's behavior, the SHAP algorithm was used to analyze the feature importance and present the contribution of each feature to the ML model's predictions (35, 36). To enhance model interpretability and trustworthiness, summary and waterfall plots were obtained to understand the model's decision-making process.

### 2.9 Application of the online prediction system

For clinical application, we developed an online warning platform that incorporates the top 10 clinically significant predictive features identified by the optimized ML models.

### 2.10 Statistical analysis

Statistical analyses were conducted using Statistical Package for the Social Sciences (SPSS) software version 26.0 (IBM Corp., Armonk, NY, USA). Continuous variables are recorded as mean (standard deviation, SD) or median (interquartile range, IQR), whereas categorical variables are expressed as *n* and percentages (*n*%). For continuous variables, comparisons were made using independent samples *t*-tests or the Mann–Whitney U-tests, depending on the data distribution. Categorical variables were assessed using the chi-squared test or Fisher's exact test, as appropriate. This study used Python programming software (version 3.12.0) (Python Software Foundation, Beaverton, United States) for data processing and model evaluation. Statistical significance was established at a *p*-value of < 0.05.

## 3 Results

### 3.1 Patient screening process

**Figure 1:**
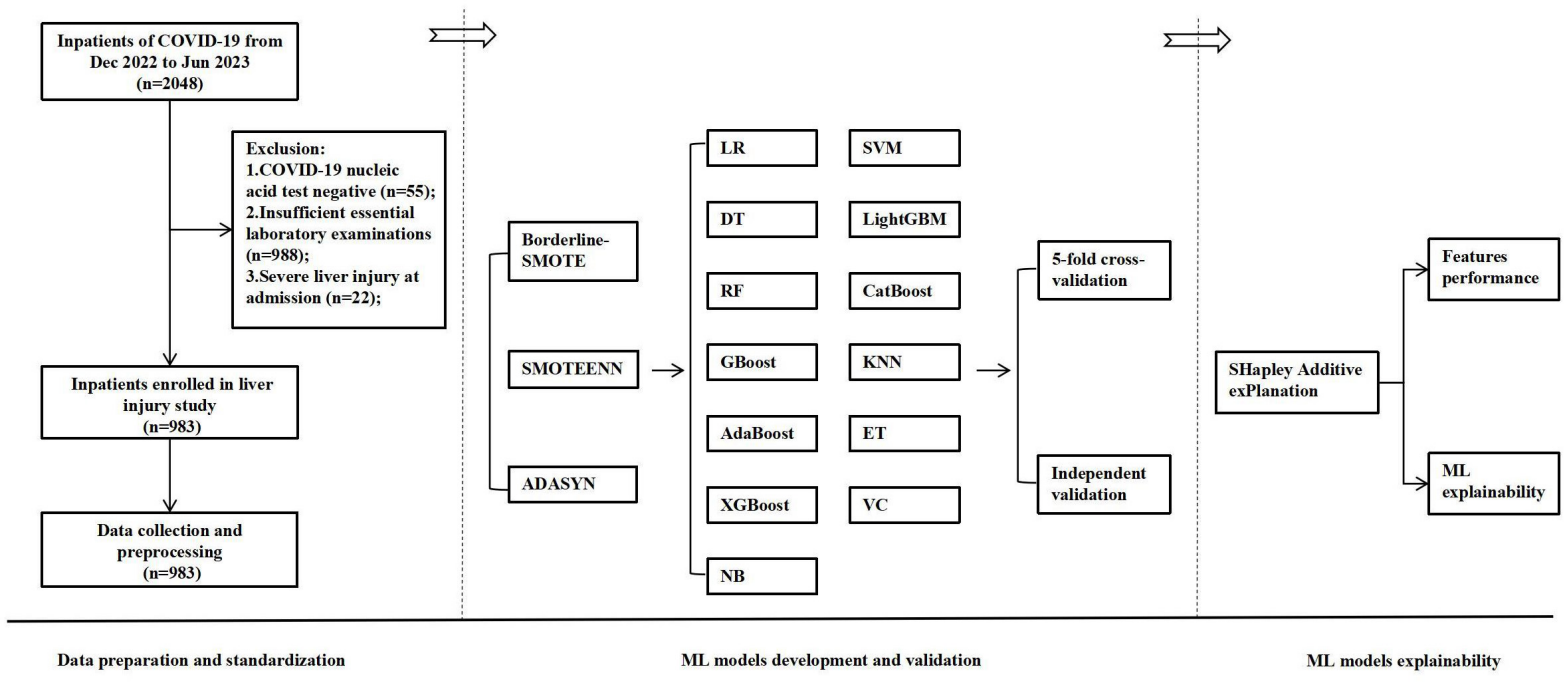
The flowchart of the developed and validated ML model-based early warning system designed for risk-stratified liver injury in hospitalized COVID-19 patients. ML, machine learning; COVID-19, coronavirus disease 2019; SMOTE, synthetic minority over-sampling technique; LR, logistic regression; DT, decision trees; RF, random forest; GBoost, gradient boosting; AdaBoost, adaptive boosting; XGBoost, eXtreme gradient boosting; NB, Naïve Bayes; SVM, support vector machine; LightGBM, light gradient boosting machine; KNN, K-nearest neighbors classifier; ET, extra trees; VC, voting classifier.

From December 2022 to June 2023, 2,048 hospitalized COVID-19 patients were identified through electronic medical records. While 55 patients (2.7%) were excluded as negative COVID-19 nucleic acid tests, 988 (48.2%) lacked laboratory tests, and 22 (1.1%) had severe liver injury at admission. Ultimately, 983 patients (48.0%) were eventually included in the analysis; the flowchart is described in Figure 1.

### 3.2 Demographic characteristics

In the COVID-19 hospital-based registry database, we collected 137 features, which included patient demographics (11 features), clinical characteristics (9 features), severity of COVID-19 (1 feature), treatment duration (1 feature), comorbidities (9 features), medications (51 features), laboratory results (54 features), infection indicators (1 feature), and an output variable (1 outcome), with detailed information provided in Supplementary Table S1. Key demographic variables include a predominance of male patients (63.28%) with a median age of 70 years (range: 59–78 years). Clinical features at admission revealed common symptoms were cough (71.82%) and fever (59.41%), while 40.49% of patients presented with severe COVID-19. The median treatment duration was 7.0 days (5.0–10.0). Prevalent comorbidities included hypertension (40.08%), heart disease (23.70%), and diabetes (19.02%). Medication use was diverse, with high frequencies of azvudine (99.49%), methylprednisolone (62.36%), and low molecular weight heparin (LMWH, 34.49%). Laboratory results showed median values of 22.0 U/L for ALT, 27.0 U/L for AST, and 61.8 g/L for total protein. Indicators of infection and inflammation, such as the percentage of neutrophils (75.3%, median) and lactate dehydrogenase (255.5 U/L, median), were elevated in a notable subset of the cohort. Additionally, 9.16% of patients experienced moderate-to-severe liver injury. Collectively, these data capture the substantial clinical and biochemical heterogeneity of hospitalized COVID-19 patients, establishing an important framework for understanding disease management strategies and outcomes in contemporary practice.

Among the 983 hospitalized COVID-19 patients, 90 (9.16%) exhibited moderate-to-severe liver injury after treatment, whereas 893(90.84%) had normal or mild results (shown in Table 1). Upon data analysis, 28 variables showed statistically significant differences between the two groups (*p* < 0.05). Patients with moderate-to-severe liver injury were more likely to be male (74.44% vs. 62.15%, *p* = 0.028) and to present with severe COVID-19 (55.56% vs. 38.97%, *p* = 0.003). Treatment duration was shorter in the liver injury group (median: 6.0 vs. 7.0 days, *p* < 0.001), and these patients received specific medications more frequently, such as doxofylline, glycyrrhizin, and glutathione (*p* < 0.05 for all). Laboratory examination revealed significantly elevated liver enzymes (e.g., ALT, AST, and GGT), bilirubin levels, and markers of inflammation (e.g., lactate dehydrogenase and neutrophil percentage) in the liver injury group (*p* < 0.01 for most), alongside decreased lymphocyte and monocyte percentages. Hematological parameters such as hemoglobin and red blood cell (RBC) were also slightly higher in this group (*p* < 0.05). It suggested that moderate-to-severe liver injury in COVID-19 was associated with greater disease severity, specific medication use, and significant changes in liver function and inflammatory markers, highlighting the need for close monitoring in such cases. Ultimately, these variables were subsequently utilized for the development and validation of ML models in our study.

**Table 1:** Primary features registered in the COVID-19 hospital-based registry database.

**Variables**	**Total (*n =* 983, 100%)**	**Non-moderate-to-severe liver injury (*n =* 893, 90.84%)**	**Moderate-to-severe liver injury (*n =* 90, 9.16%)**	***p*-value**
**Demographics**				
Male, *n* (%)	622 (63.28)	555 (62.15)	67 (74.44)	0.028^*^
**Severity of COVID-19 at admission**				
Severe, *n* (%)	398 (40.49)	348 (38.97)	50 (55.56)	0.003^**^
**Treatment duration**				
Treatment duration, day, median (range)	7.0 (5.0–10.0)	7.0 (6.0–10.0)	6.0 (4.0–7.8)	< 0.001^***^
**Medication**				
Doxofylline, *n* (%)	137 (13.94)	116 (12.99)	21 (23.33)	0.011^*^
Low molecular weight heparin, *n* (%)	339 (34.49)	297 (33.26)	42 (46.67)	0.014^*^
Diclofenac, *n* (%)	98 (9.97)	80 (8.96)	18 (20.00)	0.001^**^
Glycyrrhizin, *n* (%)	156 (15.87)	125 (14.00)	31 (34.44)	< 0.001^***^
Glutathione, *n* (%)	199 (20.24)	154 (17.25)	45 (50.00)	< 0.001^***^
Polyunsaturated phosphatidylcholine, *n* %)	30 (3.05)	15 (1.68)	15 (16.67)	< 0.001^***^
**Laboratory results**				
Alanine aminotransferase, U/L, median (range)	22.0 (14.0–35.0)	21.0 (13.0–32.0)	40.5 (24.0–70.5)	< 0.001^***^
Aspartate aminotransferase, U/L, median (range)	27.0 (19.5–39.0)	27.0 (19.0–37.0)	43.0 (28.0–70.8)	< 0.001^***^
Alkaline phosphatase, U/L, median (range)	65.0 (53.0–81.0)	64.0 (52.0–78.3)	77.0 (64.0–114.0)	< 0.001^***^
Gamma-glutamyl transferase, U/L, median (range)	29.0 (18.0–52.0)	27.0 (19.0–37.0)	73.0 (38.5–125.5)	< 0.001^***^
Total protein, g/L, median (range)	61.8 (56.6–66.0)	61.7 (56.5–66.0)	63.2 (58.4–65.9)	0.047^*^
Albumin/Globulin ratio, median (range)	1.38 (1.21–1.59)	1.39 (1.22–1.60)	1.34 (1.19–1.52)	0.028^*^
Total bilirubin, μmol/L, median (range)	9.5 (7.0–13.6)	9.40 (9.90–13.40)	11.20 (8.47–16.47)	0.003^**^
Direct bilirubin, μmol/L, median (range)	3.9 (2.7–5.6)	3.80 (2.70–5.40)	4.95 (3.40–6.72)	0.006^**^
Creatinine, U/L, median (range)	71.0 (56.0–93.0)	71.0 (56.0–93.0)	77.0 (62.3–101.0)	0.034^*^
Urea/Creatinine ratio, median (range)	13.6 (10.5–17.4)	13.5 (10.2–17.2)	15.3 (11.9–19.8)	0.014^*^
Lactate dehydrogenase, U/L, median (range)	255.5 (205.0–326.0)	253.0 (203.8–319.0)	312.5 (220.5–398.3)	0.004^**^
Neutrophil percentage, %, median (range)	75.3 (63.9–84.6)	74.8 (63.4–84.3)	81.6 (71.6–86.7)	0.006^**^
Lymphocyte percentage, %, median (range)	13.6 (7.4–21.9)	14.0 (7.5–22.1)	10.7 (6.6–16.3)	0.010^*^
Monocyte percentage, %, median (range)	7.75 (4.90–11.17)	7.90 (5.00–11.30)	6.30 (3.70–8.90)	0.005^**^
Basophil percentage, %, median (range)	0.20 (0.10–0.40)	0.20 (0.10–0.40)	0.20 (0.10–0.30)	0.029^*^
Neutrophil count, × 10^9^/L, median (range)	4.30 (2.74–6.35)	4.26 (2.71–6.26)	5.03 (3.35–8.07)	0.025^*^
Eosinophil count, × 10^9^/L, median (range)	0.02 (0.00–0.10)	0.02 (0.00–0.10)	0.00 (0.00–0.04)	0.012^*^
Red Blood cell count, × 10^12^/L, median (range)	3.98 (3.51–4.40)	3.96 (3.49–4.39)	4.16 (3.73–4.46)	0.015^*^
Hemoglobin, g/L, median (range)	121.0 (104.0–133.0)	121.0 (102.0–133.0)	127.0 (116.0–136.0)	0.019^*^

COVID-19, coronavirus disease 2019.

^*^p < 0.05; ^**^p < 0.01; ^***^p < 0.001.

### 3.3 SMOTE techniques performance

To address the class imbalance in the dataset, the diagnostic performance of five ML models (LR, DT, RF, GBoost, and KNN) was assessed using different SMOTE techniques, including Borderline-SMOTE, SMOTE-ENN, and adaptive synthetic sampling (ADASYN). The models were evaluated based on AUC, accuracy, precision, recall, and F1-score, using 5-CV (Table 2) and independent validation (Table 3).

**Table 2:** The 5-fold cross-validation diagnostic performance of the machine learning models for moderate-to-severe liver injury using different SMOTE techniques (mean ± SD).

**Model**	**AUC**	**Accuracy**	**Precision**	**Recall**	**F1-score**
**Borderline-SMOTE**					
Logistic regression (LR)	0.870 ± 0.022	0.824 ± 0.024	0.313 ± 0.033	0.750 ± 0.036	0.441 ± 0.036
Decision tree (DT)	0.642 ± 0.073	0.761 ± 0.029	0.187 ± 0.047	0.485 ± 0.147	0.269 ± 0.069
Random forest (RF)	0.835 ± 0.045	0.910 ± 0.008	0.450 ± 0.245	0.099 ± 0.073	0.160 ± 0.112
Gradient boosting (GBoost)	0.833 ± 0.071	0.914 ± 0.012	0.547 ± 0.131	0.250 ± 0.095	0.341 ± 0.113
K-Nearest neighbors classifier (KNN)	0.739 ± 0.071	0.908 ± 0.007	0.550 ± 0.245	0.124 ± 0.048	0.196 ± 0.067
**SMOTE-ENN**					
Logistic regression (LR)	0.973 ± 0.008	0.922 ± 0.017	0.929 ± 0.020	0.937 ± 0.012	0.933 ± 0.014
Decision tree (DT)	0.937 ± 0.011	0.909 ± 0.016	0.918 ± 0.017	0.924 ± 0.023	0.921 ± 0.014
Random forest (RF)	0.998 ± 0.002	0.977 ± 0.006	0.970 ± 0.009	0.992 ± 0.007	0.981 ± 0.005
Gradient boosting (GBoost)	0.997 ± 0.002	0.967 ± 0.015	0.958 ± 0.017	0.986 ± 0.010	0.972 ± 0.012
K-Nearest neighbors classifier (KNN)	0.996 ± 0.002	0.958 ± 0.011	0.932 ± 0.017	1.000 ± 0.000	0.965 ± 0.009
**ADASYN**					
Logistic regression (LR)	0.899 ± 0.032	0.835 ± 0.036	0.828 ± 0.040	0.843 ± 0.038	0.836 ± 0.037
Decision tree (DT)	0.885 ± 0.027	0.847 ± 0.022	0.813 ± 0.013	0.901 ± 0.041	0.854 ± 0.024
Random forest (RF)	0.989 ± 0.004	0.948 ± 0.021	0.929 ± 0.027	0.970 ± 0.014	0.949 ± 0.021
Gradient boosting (GBoost)	0.990 ± 0.006	0.950 ± 0.019	0.935 ± 0.024	0.968 ± 0.015	0.951 ± 0.018
K-Nearest neighbors classifier (KNN)	0.958 ± 0.014	0.869 ± 0.020	0.792 ± 0.027	1.000 ± 0.000	0.884 ± 0.016

**Table 3:** The independent validation diagnostic performance of the machine learning models for moderate-to-severe liver injury using different SMOTE techniques (mean ± SD).

**Model**	**AUC**	**Accuracy**	**Precision**	**Recall**	**F1-score**
**Borderline-SMOTE**					
Logistic regression (LR)	0.822 ± 0.032	0.817 ± 0.008	0.287 ± 0.015	0.678 ± 0.082	0.403 ± 0.028
Decision tree (DT)	0.669 ± 0.046	0.761 ± 0.042	0.199 ± 0.034	0.511 ± 0.074	0.284 ± 0.039
Random forest (RF)	0.772 ± 0.017	0.919 ± 0.012	0.620 ± 0.371	0.156 ± 0.108	0.246 ± 0.162
Gradient boosting (GBoost)	0.785 ± 0.018	0.911 ± 0.008	0.524 ± 0.111	0.244 ± 0.090	0.326 ± 0.097
K-Nearest neighbors classifier (KNN)	0.667 ± 0.038	0.911 ± 0.011	0.425 ± 0.400	0.111 ± 0.093	0.173 ± 0.148
**SMOTEENN**					
Logistic regression (LR)	0.963 ± 0.012	0.918 ± 0.017	0.936 ± 0.015	0.923 ± 0.020	0.929 ± 0.015
Decision tree (DT)	0.939 ± 0.011	0.906 ± 0.013	0.923 ± 0.017	0.914 ± 0.027	0.918 ± 0.012
Random forest (RF)	0.997 ± 0.002	0.972 ± 0.012	0.963 ± 0.011	0.990 ± 0.010	0.976 ± 0.010
Gradient boosting (GBoost)	0.994 ± 0.003	0.968 ± 0.009	0.960 ± 0.013	0.985 ± 0.007	0.972 ± 0.007
K-Nearest neighbors classifier (KNN)	0.995 ± 0.003	0.967 ± 0.002	0.947 ± 0.004	0.999 ± 0.002	0.972 ± 0.002
**ADASYN**					
Logistic regression (LR)	0.822 ± 0.032	0.817 ± 0.008	0.287 ± 0.015	0.678 ± 0.082	0.403 ± 0.028
Decision tree (DT)	0.669 ± 0.046	0.761 ± 0.042	0.199 ± 0.034	0.511 ± 0.074	0.284 ± 0.039
Random forest (RF)	0.772 ± 0.017	0.919 ± 0.012	0.620 ± 0.371	0.156 ± 0.108	0.246 ± 0.162
Gradient boosting (GBoost)	0.785 ± 0.018	0.911 ± 0.008	0.524 ± 0.111	0.244 ± 0.090	0.326 ± 0.097
K-Nearest neighbors classifier (KNN)	0.667 ± 0.038	0.911 ± 0.011	0.425 ± 0.400	0.111 ± 0.093	0.173 ± 0.148

As present in Table 2, five ML models showed moderate performance under the Borderline-SMOTE approach, with AUC values ranging from 0.642 ± 0.073 (DT) to 0.870 ± 0.022 (LR), but struggled with recall, as seen in models like RF (0.099 ± 0.073), KNN (0.124 ± 0.048), which achieved high accuracy but low recall, indicating challenges in handling imbalanced data. In contrast, the SMOTE-ENN resampling strategy significantly improved model performance, with RF, GBoost, and KNN achieving AUC over 0.995 and balanced metrics, such as recall beyond 0.98 and F1-scores exceeding 0.97, while even simpler models like LR and DT showed marked improvements in recall and F1-scores, showcasing the robustness of this technique in managing imbalanced datasets. ADASYN similarly improved performance compared to Borderline-SMOTE, but was slightly less effective than SMOTE-ENN, with RF, GBoost, and KNN, maintaining high AUC (0.958–0.990) and balanced metrics, while simpler models showed moderate gains.

Compared to the ML model's performance metrics between 5-CV and independent validation, the Borderline-SMOTE techniques showed a minimal difference between 5-fold cross-validation (5-CV) and independent validation, but the overall results were suboptimal. In RF using Borderline-SMOTE, the AUC was 0.835 ± 0.045 in 5-CV and dropped slightly to 0.772 ± 0.017 in independent validation, while the F1-score remained consistently low, at 0.160 ± 0.112 in 5-CV and 0.246 ± 0.162 in validation, respectively. This stability suggested that the models did not overfit; however, they failed to adequately handle class imbalance, resulting in poor generalization and low predictive performance. Meanwhile, ADASYN showed significant performance differences between 5-CV and independent validation, highlighting a tendency toward overfitting. For instance, RF achieved an AUC of 0.989 ± 0.004 and an F1-score of 0.949 ± 0.021 in 5-CV but dropped sharply to an AUC of 0.772 ± 0.017 and an F1-score of 0.246 ± 0.162 in validation, respectively. Similar gaps were observed for GBoost (F1-score: 0.951 ± 0.018 in 5-CV vs. 0.326 ± 0.097 in validation). These discrepancies suggest that while ADASYN improved model performance during cross-validation, it led to overfitting by relying too heavily on synthetic samples, thereby reducing the model's generalizability to unseen data.

In contrast, SMOTE-ENN demonstrated consistent performance across 5-CV and independent validation, highlighting its robustness and ability to generalize effectively. For RF, the AUC was 0.998 ± 0.002 in 5-CV and 0.997 ± 0.002 in validation, with the F1-score remaining high at 0.981 ± 0.005 and 0.976 ± 0.010, respectively. Similar trends were observed with KNN, where the AUC was 0.996 ± 0.002 in 5-CV and 0.995 ± 0.003 in validation, with F1-scores of 1.000 ± 0.000 and 0.999 ± 0.002, respectively. This consistency demonstrated that SMOTE-ENN effectively addressed class imbalance without introducing overfitting, making it the most reliable resampling technique for this purpose. Based on the above analysis, SMOTE-ENN was selected to address data imbalance and develop ML models.

### 3.4 ML models' performance

**Figure 2:**
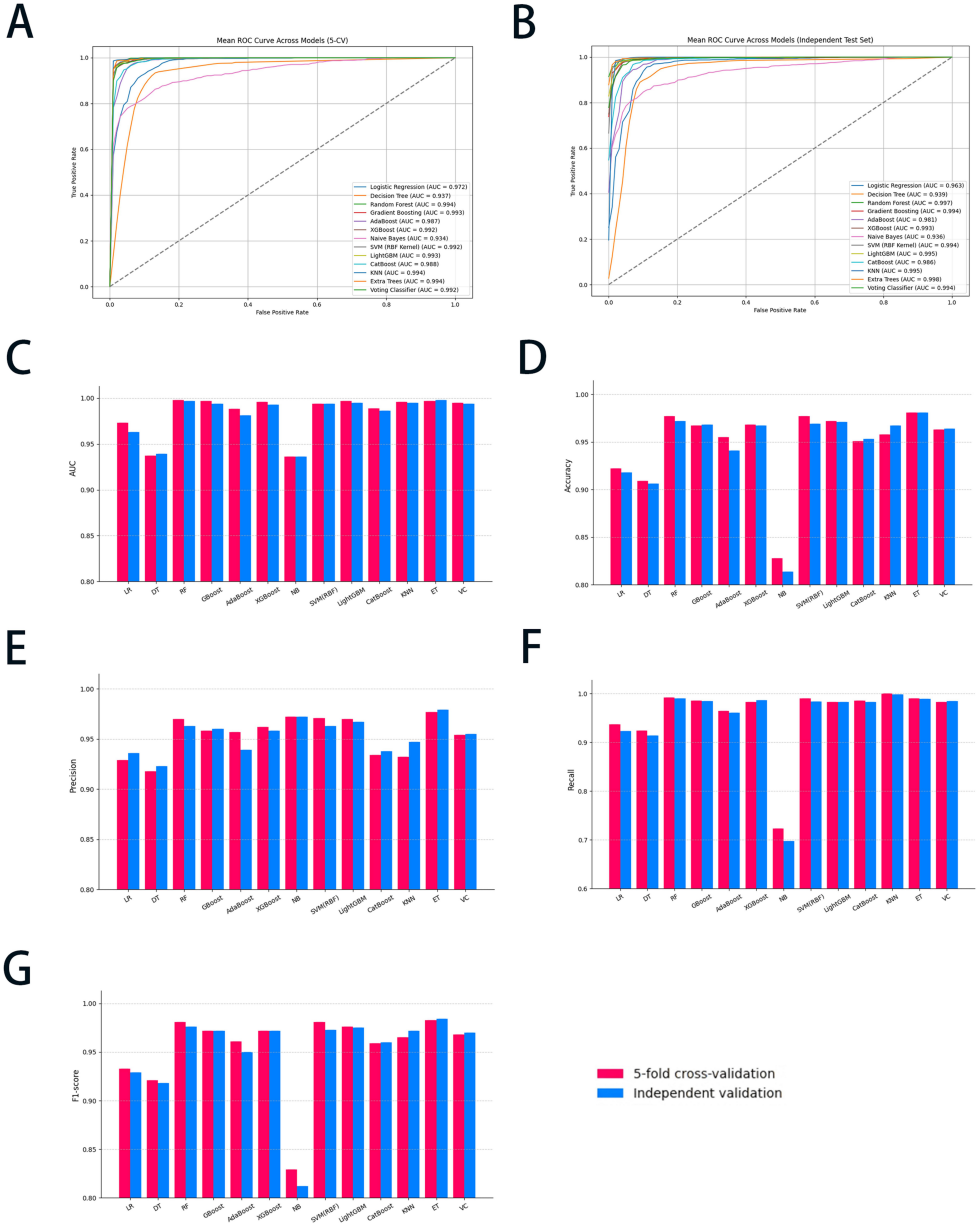
The diagnostic performance of all ML models with 5-CV and independent validation. **(A)** The ROC curve of the 5-CV; **(B)** the ROC curve of the independent validation; **(C)** the AUC value of all ML models; **(D)** the accuracy of all ML models; **(E)** the precision of all ML models; **(F)** the recall of all ML models; and **(G)** the F1-score of all ML models. ML, machine learning; 5-CV, 5-fold cross-validation; ROC, receiver operating characteristic curve; AUC, area under the curve; LR, logistic regression; DT, decision trees; RF, random forest; GBoost, gradient boosting; AdaBoost, adaptive boosting; XGBoost, eXtreme gradient boosting; NB, Naïve Bayes; SVM, support vector machine; LightGBM, light gradient boosting machine; KNN, K-nearest neighbors classifier; ET, extra trees; VC, voting classifier.

All thirteen ML algorithms achieved substantial and consistent performance (Figure 2), demonstrating the technique's effectiveness in addressing data imbalance and improving model generalization. Among the tested algorithms, RF and ET emerged as the top performers. RF achieved a high AUC of 0.998 ± 0.002 in 5-CV, while recall obtained with 0.992 ± 0.007, respectively. ET slightly underperformed RF with an AUC of 0.997 ± 0.002 and a recall of 0.990 ± 0.013 in 5-CV. Both models demonstrated high F1-score, with RF achieving 0.981 ± 0.005 and ET 0.983 ± 0.011, respectively, highlighting their superior ability to classify minority-class instances correctly. In the meantime, GBoost achieved an AUC of 0.997 ± 0.002 and an F1-score of 0.972 ± 0.012, demonstrating strong overall performance, though slightly behind the tree-based models. Similarly, LightGBM achieved an AUC of 0.997 ± 0.002 and an F1-score of 0.976 ± 0.008, with metrics comparable to GBoost. These algorithms, while strong, did not consistently match the precision and recall of ET and RF. SVM (using a radial basis function kernel [RBF kernel]) and VC showed competitive results, but lagged slightly behind the top-performing models. SVM (RBF kernel) achieved an AUC of 0.994 ± 0.004 and an F1-score of 0.981 ± 0.008, maintaining a strong balance between sensitivity and specificity. The VC, which combines predictions from multiple algorithms, achieved an AUC of 0.995 ± 0.003 and an F1-score of 0.968 ± 0.011, but it did not surpass the tree-based models in recall or overall performance. In addition, no significant differences in data were observed when comparing 5-CV with independent validation across all algorithms, indicating that the model possesses satisfactory robustness and specificity. In summary, while SMOTE-ENN improved the performance of all models, RF and ET emerged as the most effective algorithms and were selected as the ML algorithms for the study.

### 3.5 Features performance

**Figure 3:**
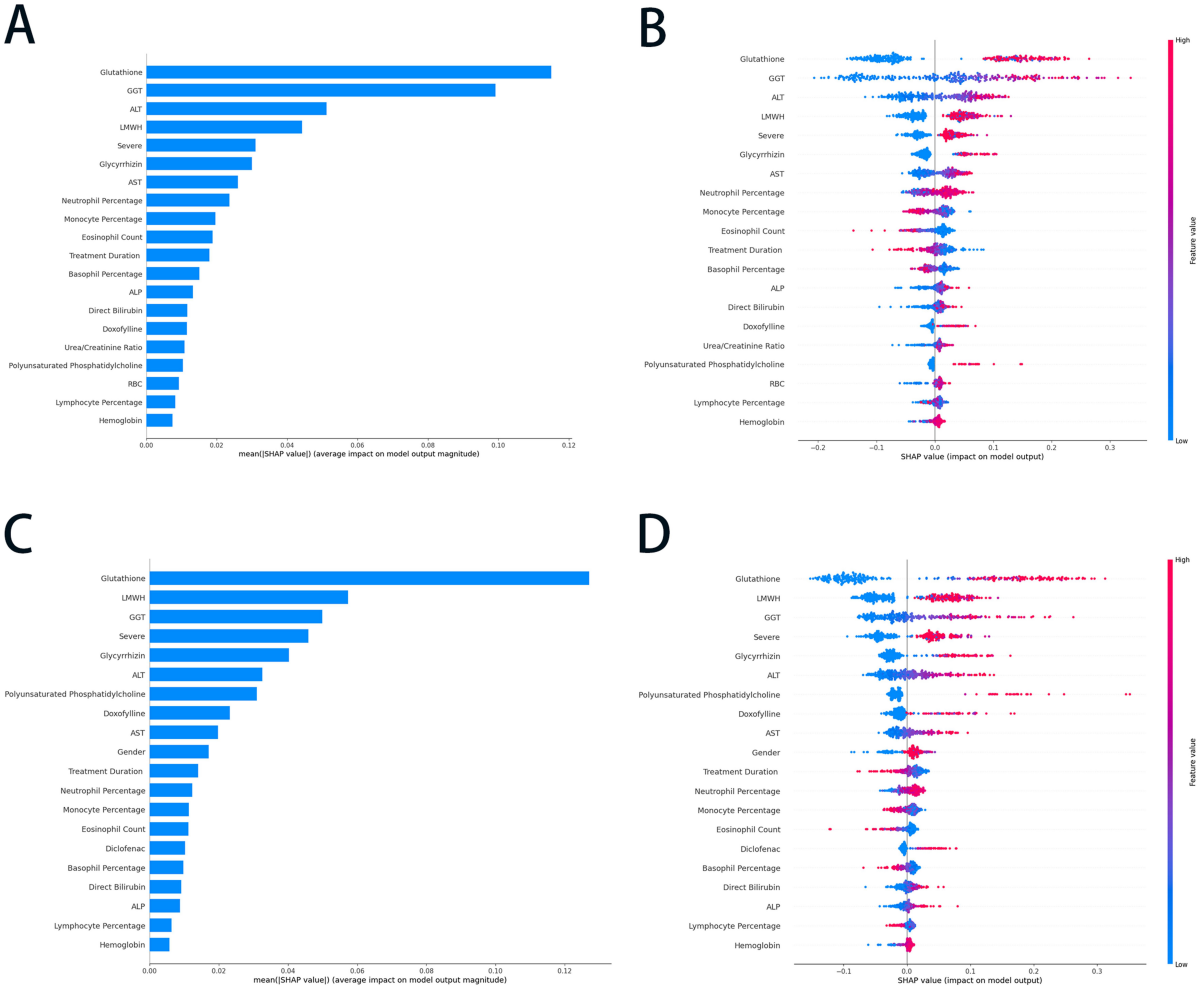
The SHAP summary plots of the RF and ET models. **(A)** The average SHAP values of the top 20 features in the RF model; **(B)** the SHAP values (impact on model output) of the top 20 features in the RF model; **(C)** the average SHAP values of the top 20 features in ET model; and **(D)** the SHAP values (impact on model output) of the top 20 features in ET model. SHAP, SHapley Additive exPlanations; RF, random forest; ET, extra trees; GGT, gamma-glutamyltransferase; ALT, alanine aminotransferase; AST, aspartate transaminase; LMWH, Low molecular weight heparin; ALP, alkaline phosphatase; RBC, red blood cell.

The SHAP summary plots (Figure 3) offered an in-depth analysis of feature importance and their contributions to the predictive performance of RF and ET models. In Figure 3A, features were ranked according to their average SHAP values, which reflect their overall impact on the RF model's output. The use of glutathione, GGT, ALT, and low molecular weight heparin (LMWH) exhibited the highest contributions, indicating their significant predictive influence. In contrast, features such as hemoglobin and lymphocyte percentage demonstrated relatively minimal impact. Figure 3B provided a nuanced representation of each feature's contribution, with SHAP values indicating the magnitude and direction of their effect on RF model predictions. The color gradient highlights feature values, where red denotes higher values, and blue represents lower values. For instance, elevated levels of glutathione and GGT were associated with a positive influence on predictions. At the same time, lower values of features such as neutrophil percentage and treatment duration exerted a negative impact. These plots underscore the pivotal role of biochemical markers and clinical parameters in shaping the RF model's predictive outcomes.

In the ET model, glutathione, LMWH, GGT, and severe were identified as the most influential predictors, indicating their critical role in driving the model's output (Figure 3C). Conversely, lymphocyte percentage, ALP, and hemoglobin exhibited minimal average SHAP values, suggesting their limited predictive relevance. Figure 3D provided a more granular perspective; higher values of glutathione, LMWH, and GGT were associated with positive contributions to predictions, whereas lower values of neutrophil percentage, monocyte percentage, and eosinophil count had a negative influence on the outcomes. Features such as treatment duration, glycyrrhizin, and AST demonstrated moderate contributions, further underscoring their relevance. These plots highlighted the pivotal roles of specific biochemical markers and clinical parameters in shaping the ET model's predictions, showcasing its reliance on key features for accurate and reliable outcomes.

### 3.6 ML models explainability

**Figure 4:**
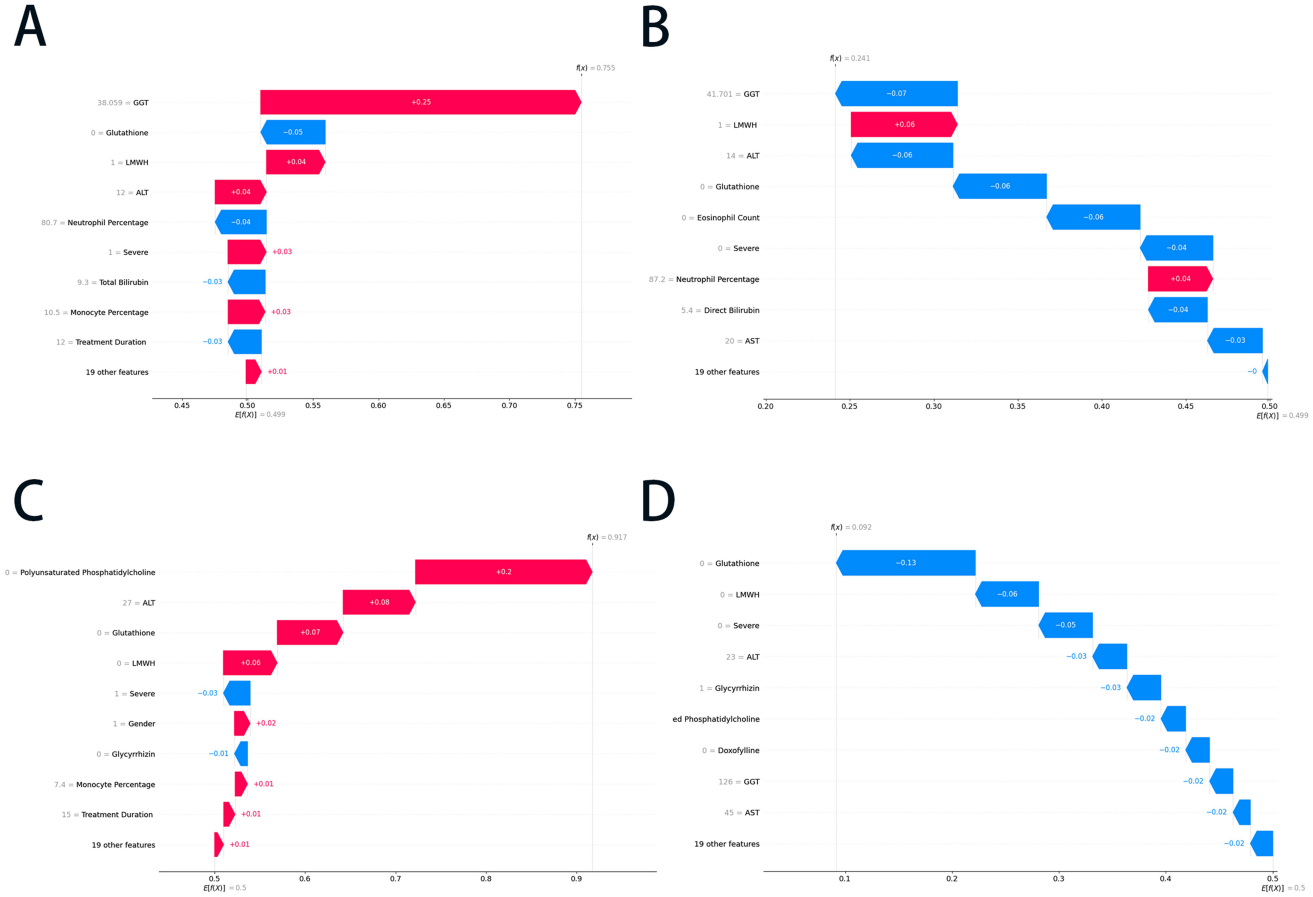
The SHAP waterfall plots of the RF and ET models. **(A)** The positive prediction case in the RF model; **(B)** the negative prediction case in the RF model; **(C)** the positive prediction case in the ET model; and **(D)** the negative prediction case in the ET model. SHAP, SHapley Additive exPlanations; RF, random forest; ET, extra trees; GGT, gamma-glutamyltransferase; ALT, alanine aminotransferase; AST, aspartate transaminase; LMWH, low molecular weight heparin.

The SHAP waterfall plots shown in Figures 4A, B illustrate the RF model's prediction of liver injury risk for two patients based on key features. Figure 4A showed a high-risk prediction with a final SHAP value of 0.755, mainly due to GGT (+0.25), LMWH use (+0.04), and ALT (+0.04). Other factors, such as severe (+0.03) and monocyte percentage (+0.03), shifted the base value to a high-risk outcome. In contrast, Figure 4B depicts a low-risk prediction with a final SHAP value of 0.241, driven by normal GGT (−0.07), lower ALT (−0.06), and the absence of glutathione (−0.06) use. Additional negative influences, including a lower eosinophil count (−0.06) and non-severe conditions (−0.04), decreased the risk, while eliminating positive contributions of LMWH use (+0.06) and higher neutrophil percentages (+0.04). The above prediction results were consistent with the actual outcome (Supplementary Table S2), providing a reference for clinical decision-making.

Figures 4C, D show the ET model's predictions. Figure 4C indicates a positive prediction with a final SHAP value of 0.917, primarily driven by the absence of polyunsaturated phosphatidylcholine (+0.20) and glutathione (+0.08), with minor contributions from LMWH (+0.06) and gender (+0.02). Negative contributors, such as those with a severe (−0.03) rating, slightly offset the risk. Figure 4D presented a negative prediction with a final SHAP value of 0.092, characterized by strong negative contributions from the absence of glutathione, LMWH, and severe disease, along with low levels of ALT, GGT, and AST. These plots demonstrated the ET model's integration of positive and negative feature contributions, emphasizing the role of liver function markers, treatment factors, and clinical severity in risk stratification. The predictions in the cases above corresponded with the actual clinical outcomes (Supplementary Table S2), demonstrating that the ET model achieves high accuracy.

### 3.7 Application of the ML models

**Figure 5:**
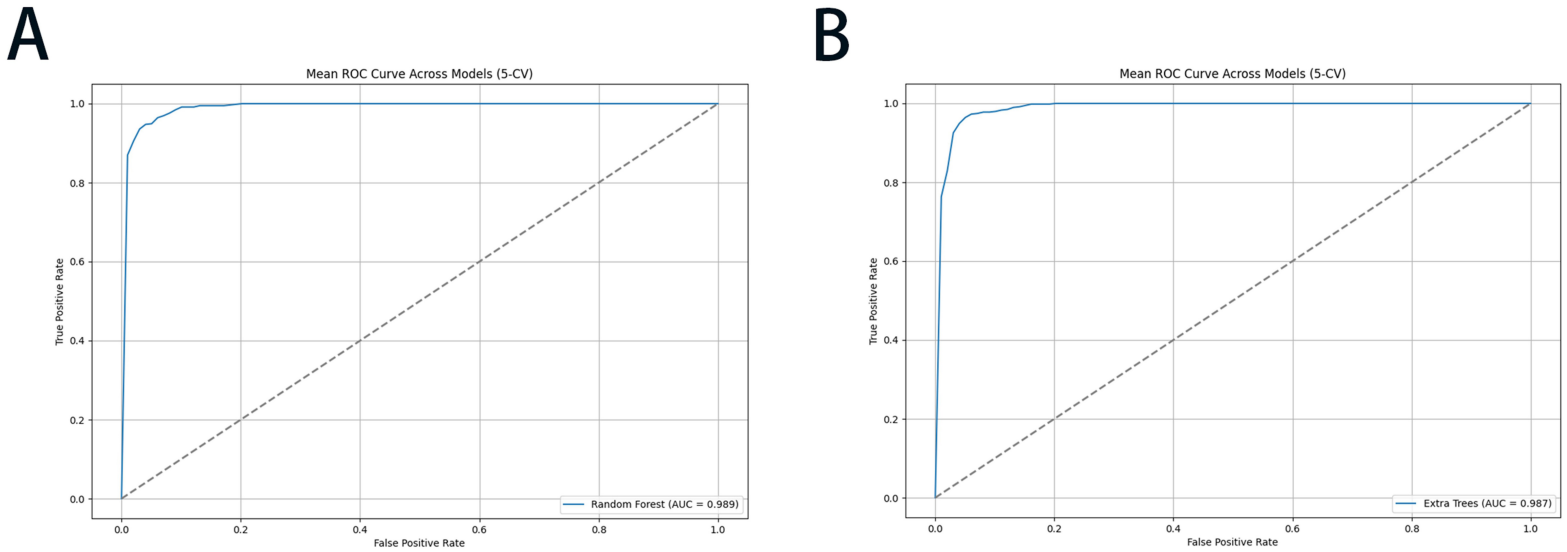
The ROC performance of the RF and ET models with 5-CV for risk-stratified liver injury prediction: **(A)** RF; **(B)** ET. ROC, receiver operating characteristic curve; RF, random forest; ET, extra trees; 5-CV, 5-fold cross-validation.

To streamline the practical implementation of our validated models, we prioritized the top 10 ranked feature factors for developing a simplified online prediction system. The constructed RF and ET models demonstrated robust predictive performance, with AUC values of 0.994 ± 0.003 and 0.992 ± 0.004, respectively. The corresponding ROC curves were graphically presented in Figure 5, while comprehensive model parameters were systematically documented in Supplementary Table S3. This optimized model enables the early warning system for risk-stratified liver injury prediction through a web-based interface by incorporating relevant clinical features of individual patients. The online prediction system was publicly accessible at the URLs https://10jarg4450699.vicp.fun (RF model) and http://10jarg4450699.vicp.fun (ET model).

## 4 Discussion

This study developed and validated an innovative, early risk-stratified system for predicting the progression of liver injury in COVID-19 hospitalized patients. The diagnostic ML models for system development demonstrated accuracy in both the training and validation cohorts, and their performance remained stable across different subgroups. To the best of our knowledge, this represents the pioneering effort to apply real-world data within a predictive framework, employing a categorical methodology to stratify the severity of hepatic dysfunction. This optimized prediction system demonstrates significant potential as an instrumental tool, providing clinicians with a sophisticated reference for the meticulous management of patient care and the formulation of targeted therapeutic strategies.

Liver dysfunction has been widely considered a common complication among patients with COVID-19, while various studies have highlighted its prevalence and clinical significance. Previous studies have documented that hepatocellular injury manifests in 14–53% of COVID-19 patients, typically characterized by an elevation in aminotransferase levels below 5-fold ULN in the initial stage of the pandemic (37). We found that 51.2% of patients had a liver injury on admission, increasing to 70.0% during hospitalization in an earlier stage of COVID-19 (38). Recently, Ruveena et al. conducted a multicenter study enrolling 1,246 hospitalized patients and confirmed that ~58.7% of patients presented with abnormal liver biochemistry, and 47.7% had persistent abnormalities up to 6 months after infection (39). Furthermore, to elucidate the distinctions among various strains in the context of liver injury, we conducted an extensive evaluation of hepatic function involving 1,420 patients during the Omicron wave of the pandemic. Our findings revealed that abnormal liver function was observed during hospitalization in 45.2% (444/983) cases; of these, 354 cases were grade 1, 63 were grade 2, and 27 were grade 3 or above, with the main manifestations being elevated levels of ALT, AST, and GGT, respectively. It is noteworthy that liver injury in COVID-19 patients is prevalent and persists throughout hospitalization, unaffected by viral mutations. This aspect should be taken into consideration in clinical management.

Based on clinical guidelines, grade 1 liver injury was generally considered clinically insignificant. Consequently, our study focused on more severe hepatic dysfunction (grade 2+). This clinically justified approach resulted in a selection bias, with only 90 positive cases (90/983) among 983 eligible patients, yielding a distribution ratio of 1:9.2. To address this fundamental challenge in ML applications, we implemented and compared three advanced resampling techniques, Borderline-SMOTE, SMOTE-ENN, and ADASYN. After applying these techniques, the class distribution was substantially balanced. Specifically, Borderline-SMOTE and ADASYN increased the minority class representation to 50.0% (893/1,786), while SMOTE-ENN achieved a minority class percentage of 57.8% (893/1,545), respectively.

The differences in the performance of various SMOTE techniques could be explained by the approaches to handling class imbalance, noise, and overfitting (40). Borderline-SMOTE partially generates synthetic samples near the decision boundary, improving recall for misclassified samples. However, it failed to address noisy or overlapping samples from the majority class, which limited its effectiveness and resulted in only marginal performance gains, as seen in GBoost's F1-score of 0.326 ± 0.097 in validation (41). The ADASYN algorithm focuses on underrepresented synthetic samples in regions where the minority class is most underrepresented. While enhancing cross-validation performance, it led to overfitting in our research. The synthetic samples generated were too tailored to the training set, causing poor generalization to unseen data (42). For instance, the F1-score of RF dropped significantly from 0.949 ± 0.021 in 5-CV to 0.246 ± 0.162 in independent validation, highlighting the issue of overfitting. In contrast, the SMOTE-ENN algorithm demonstrated superior efficacy by combining SMOTE's ability to generate balanced, synthetic samples with ENN's noise reduction, which removed noisy majority-class samples near the decision boundary (31). This dual combination produced cleaner, more balanced datasets, allowing models to learn robust decision boundaries. SMOTE-ENN consistently delivered superior performance across all metrics, with RF achieving an F1-score of 0.976 ± 0.010 and ET achieving 0.984 ± 0.006, respectively. The SMOTE-ENN's balance of oversampling and noise removal ensured generalization, making it the most effective technique in this study (43). These findings underscore the crucial role of advanced resampling strategies in enhancing the diagnostic performance of ML models for imbalanced medical datasets, underscoring their importance in clinical decision-making applications.

In this retrospective study, the predictive performance of multiple ML algorithms was evaluated. Among the assessed models, the RF and ET algorithms outperformed simpler models such as LR and NB. The RF algorithm, which utilizes a bootstrapping approach to aggregate outputs from multiple decision trees, demonstrated the highest predictive accuracy. Similarly, the ET algorithm effectively captured complex interactions between variables, highlighting its suitability for clinical prediction tasks. The potential reasons for the superior performance of RF and ET are as follows: Firstly, RF and ET excelled at capturing complex non-linear relationships and variable interactions, making them highly effective in medical prediction tasks; Secondly, they demonstrated strong robustness to noise and missing data, while being well-suited for high-dimensional, small-sample datasets; Finally, their ability to evaluate feature importance enhanced interpretability, providing valuable insights for clinical decision-making (30, 44, 45). These strengths established RF and ET as indispensable tools for managing the complexities of medical data and improving predictive accuracy in healthcare applications.

In our study, we integrated the complex interplay between liver function, kidney function, inflammatory markers, and medication factors into ML algorithms. This approach significantly enhanced the accuracy of liver injury prediction and demonstrated potential value for clinical trials. The SHAP plots for both the RF and ET models highlighted several key factors contributing to the prediction of liver injury in COVID-19 patients. Within the top ten features identified in these models, three primary categories of indicators emerged: hepatic enzyme indexes (e.g., ALT, AST, and GGT), infectious indexes (e.g., neutrophil percentage, monocyte percentage, and eosinophil count), and medications (e.g., glutathione, glycyrrhizin, phosphatidylcholine, LMWH, and doxophylline). Previous studies reported that hepatocellular damage in COVID-19 patients was likely induced by medications, a systemic inflammatory response, and the sequelae of hypoxia-ischemia reperfusion injury (46–48). Initially, within the clinical environment, the potential hepatotoxic effects of medications metabolized in the liver, such as lopinavir, ritonavir, and remdesivir, had to be considered in the context of liver injury (48, 49, 63). Subsequently, the SARS-CoV-2 virus may elicit a hyperinflammatory state by triggering an excessively robust immune response, leading to substantial tissue devastation (50, 62). This pathological process could impair pulmonary and hepatic function, partially due to the high expression of angiotensin-converting enzyme 2 (ACE2) receptors in these organs (37, 51). Moreover, elevated hepatic enzyme levels indicated that preexisting liver injury had led to a higher degree of hepatocellular damage, which might be attributed to the fact that the compromised liver was in a state of overload and more susceptible to further injury (39). Elevated liver enzymes, such as GGT, ALT, and AST, highlighted the direct relationship between liver function markers and liver injury. For instance, GGT, an enzyme associated with bile duct and liver function, had been identified as a significant predictor of liver injury in COVID-19 and was confirmed as such in our study (52). Disease severity was another prominent factor, as patients with more severe conditions were more likely to experience significant liver injury due to factors such as systemic inflammation, hypoxia, and multiorgan involvement (48, 53). These results suggested that both liver-specific factors and systemic influences, including medication effects and disease severity, were critical in predicting liver injury in COVID-19 patients.

While existing ML approaches for predicting hepatic toxicity have provided important mechanistic insights through modeling, significant translational limitations persist (60). There were substantial translational challenges due to the discordance between preclinical drug databases and real-world clinical trajectories (54, 55). Our research represented the first attempt to develop predictive models to integrate liver-toxic and liver-protective medications with real-world data. This innovation allowed clinicians to evaluate patients' pharmacological treatment plans and implement individualized therapies, thereby enabling more targeted medical monitoring. LMWH, an anticoagulant frequently administered in severe COVID-19 cases, exhibited a strong association with liver injury. This correlation likely stemmed from its systemic effects on inflammation and liver function in critically ill patients (56, 57). A case-control study involving 2,141 COVID-19 patients revealed that the co-administration of LMWH was a statistically significant risk factor for liver injury (58). Similarly, in patients with pulmonary embolism treated with LMWH, 17.1% exhibited liver dysfunction (57). Interestingly, doxophylline (DOXO), a bronchodilator commonly used to treat pulmonary diseases, has also been identified as a predictor of liver injury. This may be attributed to its hepatic metabolism, which was sensitive to the stimulation or inhibition of the P450 enzyme system. Thus, the dosage of DOXO could be increased by various drugs, including corticosteroids, macrolides, and quinolone antibiotics (51). Furthermore, DOXO might influence the metabolism of other drugs, potentially contributing to liver injury. On the other hand, our ML models highlighted liver-protective properties as influential factors. For instance, glutathione, a key antioxidant involved in liver detoxification, consistently ranked highly. This underscored its crucial role in maintaining liver function and its association with oxidative stress, a common feature in COVID-19 (10, 59). It was noteworthy that the use of liver-protective drugs in high-risk patients still led to a higher level of liver damage, indicating that the treatment method required further optimization, such as the combination with other types of detoxification drugs.

Overall, our AI-driven framework facilitated timely risk stratification by performing continuous multivariate pattern analysis of electronic health records (EHRs), thereby triggering protocolized confirmatory retesting and reducing diagnostic delays in the detection of subclinical hepatotoxicity. Our study advances clinical management in three key ways. First, the robust model training on real-world data. The ML models were trained on diverse, representative EHRs, capturing a wide spectrum of clinical characteristics to ensure accurate and reliable predictions. Second, non-invasive and cost-effective monitoring. By leveraging routinely collected EHR data, our approach reduces reliance on traditional liver function testing, minimizing healthcare costs and patient burden. Thirdly, data-driven clinical decision support. The model's predictive capabilities provide evidence-based guidance for personalized interventions, mitigating hepatotoxicity, personalized medicine, reducing liver risks, and improving patient outcomes.

While our research demonstrated promising results, several limitations must be acknowledged. First, as a retrospective study, it was inherently subject to biases, which may have led to the omission of specific crucial parameters. For instance, data on body mass index (BMI) and mechanical ventilation were not recorded in the medical records and, therefore, could not be included. Second, the study population was predominantly of East Asian descent, with no representation from other ethnic groups, which made the generalizability of the findings to other populations uncertain. Third, as a single-center study, it lacked external validation. Therefore, further prospective multicenter studies were needed to confirm and extend the applicability of these findings.

## 5 Conclusion

In this study, we developed an ML model-based early warning system for risk-stratified liver injury in hospitalized COVID-19 patients with real-world data from a large cohort. The system was trained with multiple ML models, with RF and ET emerging as the most effective in accurately predicting early-stage (moderate-to-severe) cases of liver injury. The system demonstrated strong predictive capability by integrating various clinical features, such as liver function tests, inflammation markers, and medication history. SHAP analysis revealed key features influencing the system model's predictions, including glutathione levels, GGT, LMWH use, and disease severity, all of which were significantly associated with liver injury risk. This system provides valuable decision support for clinicians, enabling the early identification of patients at risk and facilitating tailored therapeutic interventions. This study highlighted the potential of ML algorithms to enhance clinical decision-making and improve outcomes for COVID-19 patients with liver complications.

## Data Availability

The original contributions presented in the study are included in the article/Supplementary material, further inquiries can be directed to the corresponding authors.
