# Supplementary material for: Real-world data-driven early warning system for risk-stratified liver injury in hospitalized COVID-19 patients—Machine learning models for clinical decision support
Source: Front Public Health. 2025 Sep 3;13:1566260. doi: 10.3389/fpubh.2025.1566260 (PMC12441008; doi:10.3389/fpubh.2025.1566260)
Supplement: Supplementary file 1 [file Data_Sheet_1.doc]

**Supplement Table 1.** Baseline characteristics of the cohort in the COVID-19 hospital-based registry database.

| **No.** | **Features name** | **Variable type** | **Overall (n= 983)** | **No.** | **Features name** | **Variable type** | **Overall (n= 983)** |
| --- | --- | --- | --- | --- | --- | --- | --- |
| Demographics | | | | 69 | Dapagliflozin, n(%) | Categorical | 36(3.66) |
| 1 | Male, n(%) | Categorical | 622(63.28) | 70 | Dexamethasone, n(%) | Categorical | 299(30.42) |
| 2 | Age, year, median (range) | Continuous | 70.0(59.0-78.0) | 71 | Methylprednisolone, n(%) | Categorical | 613(62.36) |
| 3 | Weight, kg, median (range) | Continuous | 65.0(58.0-72.5) | 72 | Hydrocortisone, n(%) | Categorical | 31(3.15) |
| 4 | Temperature, ℃, median (range) | Continuous | 36.5(36.3-36.7) | 73 | Pantoprazole, n(%) | Categorical | 249(25.33) |
| 5 | Heart rate, bpm, median (range) | Continuous | 85.0(77.8-96.0) | 74 | Rabeprazole, n(%) | Categorical | 301(30.62) |
| 6 | Respiratory rate, bpm, median (range) | Continuous | 19.0(18.0-20.0) | 75 | Esomeprazole, n(%) | Categorical | 61(6.21) |
| 7 | Systolic Blood Pressure, mmHg, median (range) | Continuous | 125.0(110.0-138.0) | 76 | Omeprazole, n(%) | Categorical | 152(15.46) |
| 8 | Diastolic Blood Pressure, mmHg, median (range) | Continuous | 76.0(69.0-87.0) | 77 | Esomeprazole, n(%) | Categorical | 35(3.56) |
| 9 | Smoke, n(%) | Categorical | 95(9.66) | 78 | Diclofenac, n(%) | Categorical | 98(9.97) |
| 10 | Drink, n(%) | Categorical | 57(5.80) | 79 | Ibuprofen, n(%) | Categorical | 37(3.76) |
| 11 | Allergic, n(%) | Categorical | 74(7.53) | 80 | Glycyrrhizin, n(%) | Categorical | 156(15.87) |
| Clinical features at admission | | | | 81 | Gutathione, n(%) | Categorical | 199(20.24) |
| 12 | Fever, n(%) | Categorical | 584(59.41) | 82 | Polyunsaturated Phosphatidylcholine, n(%) | Categorical | 30(3.05) |
| 13 | Cough, n(%) | Categorical | 706(71.82) | Laboratory results | | | |
| 14 | Sputum, n(%) | Categorical | 559(56.87) | 83 | Alanine aminotransferase, U/L, median (range) | Continuous | 22.0(14.0-35.0) |
| 15 | Chest tightness, n(%) | Categorical | 277(28.18) | 84 | Aspartate aminotransferase, U/L, median (range) | Continuous | 27.0(19.5-39.0) |
| 16 | Fatigue, n(%) | Categorical | 354(36.01) | 85 | ALT/AST ratio, median (range) | Continuous | 0.80(0.60-1.11) |
| 17 | Dyspepsia, n(%) | Categorical | 263(26.75) | 86 | Alkaline phosphatase, U/L, median (range) | Continuous | 65.0(53.0-81.0) |
| 18 | Nausea, n(%) | Categorical | 46(4.68) | 87 | Gamma-glutamyl transferase, U/L, median (range) | Continuous | 29.0(18.0-52.0) |
| 19 | Vomiting, n(%) | Categorical | 40(4.07) | 88 | Total bile acids, μmol/L, median (range) | Continuous | 3.59(2.08-7.06) |
| 20 | Diarrhoea, n(%) | Categorical | 41(4.17) | 89 | Prealbumin, g/L, median (range) | Continuous | 105.2(64.0-156.2) |
| Severity of COVID-19 at admission | | | | 90 | Total Protein, g/L, median (range) | Continuous | 61.8(56.6-66.0) |
| 21 | Severe, n(%) | Categorical | 398(40.49) | 91 | Albumin, g/L, median (range) | Continuous | 35.5(32.4-38.4) |
| Treatment Duration | | | | 92 | Globulin, g/L, median (range) | Continuous | 26.1(22.8-29.0) |
| 22 | Treatment duration, day, median (range) | Continuous | 7.0(5.0-10.0) | 93 | Albumin/Globulin Ratio, median (range) | Continuous | 1.38(1.21-1.59) |
| Commodities | | | | 94 | Total Bilirubin, μmol/L, median (range) | Continuous | 9.5(7.0-13.6) |
| 23 | Liver disease, n(%) | Categorical | 112(11.39) | 95 | Direct Bilirubin, μmol/L, median (range) | Continuous | 3.9(2.7-5.6) |
| 24 | Kidney disease, n(%) | Categorical | 202(20.55) | 96 | Cholinesterase, U/L, median (range) | Continuous | 6233.0(4438.5-7794.0) |
| 25 | Hypertension, n(%) | Categorical | 394(40.08) | 97 | Urea, U/L, median (range) | Continuous | 6.50(4.86-9.27) |
| 26 | Heart Disease, n(%) | Categorical | 233(23.70) | 98 | Creatinine, U/L, median (range) | Continuous | 71.0(56.0-93.0) |
| 27 | Diabetes, n(%) | Categorical | 187(19.02) | 99 | Urea/Creatinine Ratio, median (range) | Continuous | 13.6(10.5-17.4) |
| 28 | Cancer, n(%) | Categorical | 142(14.45) | 100 | Total Carbon Dioxide, mmol/L, median(range) | Continuous | 25.1(23.0-27.2) |
| 29 | Stroke, n(%) | Categorical | 80(8.14) | 101 | Serum Uric Acid, μmol/L, median(range) | Continuous | 266.0(201.0-346.0) |
| 30 | Thyroid Disease, n(%) | Categorical | 58(5.90) | 102 | Serum Glucose, mmol/L, median(range) | Continuous | 6.72(5.40-8.57) |
| 31 | Nutrition, n(%) | Categorical | 197(20.04) | 103 | Potassium, mmol/L, median(range) | Continuous | 3.99(3.63-4.39) |
| Medication | | | | 104 | Sodium, mmol/L, median(range) | Continuous | 139.5(137.0-141.7) |
| 32 | Azvudine, n(%) | Categorical | 978(99.49) | 105 | Chloride, mmol/L, median(range) | Continuous | 106.3(103.1-108.9) |
| 33 | Ganciclovir, n(%) | Categorical | 111(11.29) | 106 | Calcium, mmol/L, median(range) | Continuous | 2.09(1.97-2.20) |
| 34 | Nirmatrelvir/Ritonavir & Paltamirvir, n(%) | Categorical | 33(3.36) | 107 | Corrected Calcium, mmol/L, median(range) | Continuous | 2.17(2.10-2.23) |
| 35 | Cefmetazole, n(%) | Categorical | 121(12.31) | 108 | Magnesium, mmol/L, median(range) | Continuous | 0.81(0.74-0.87) |
| 36 | Cefaclor, n(%) | Categorical | 35(3.56) | 109 | Inorganic Phosphate, mmol/L, median(range) | Continuous | 1.00(0.84-1.19) |
| 37 | Cefoperazone/Sulbactam, n(%) | Categorical | 340(34.59) | 110 | Calcium-Phosphorus Product, mmol^2^/L^2^, median(range) | Continuous | 2.39(1.99-2.82) |
| 38 | Ceftriaxone, n(%) | Categorical | 153(15.56) | 111 | Anion Gap, mmol/L, median(range) | Continuous | 12.1(10.6-14.0) |
| 39 | Ceftazidime, n(%) | Categorical | 188(19.13) | 112 | Osmolality, mosm/L, median(range) | Continuous | 301.6(296.3-307.3) |
| 40 | Levofloxacin, n(%) | Categorical | 88(8.95) | 113 | Total Cholesterol, mmol/L, median(range) | Continuous | 3.65(3.15-4.31) |
| 41 | Moxifloxacin, n(%) | Categorical | 395(40.18) | 114 | Triglycerides, mmol/L, median(range) | Continuous | 1.21(0.91-1.63) |
| 42 | Meropenem, n(%) | Categorical | 86(8.75) | 115 | Serum HDL Cholesterol, mmol/L, median(range) | Continuous | 0.85(0.67-1.09) |
| 43 | Imipenem, n(%) | Categorical | 67(6.82) | 116 | Serum LDL Cholesterol, mmol/L, median(range) | Continuous | 2.20(1.66-2.79) |
| 44 | Teicoplanin, n(%) | Categorical | 32(3.26) | 117 | Total/HDL Cholesterol, median(range) | Continuous | 4.29(3.51-5.34) |
| 45 | Voriconazole, n(%) | Categorical | 39(3.97) | 118 | Serum Creatine Kinase, U/L, median(range) | Continuous | 63.0(38.0-115.0) |
| 46 | Doxofylline, n(%) | Categorical | 137(13.94) | 119 | Lactate Dehydrogenase, U/L, median(range) | Continuous | 255.5(205.0-326.0) |
| 47 | Ambroxol, n(%) | Categorical | 407(41.40) | 120 | Estimated glomerular filtration rate, mL/min, median(range) | Continuous | 81.1(51.1-94.2) |
| 48 | Methoxamine, n(%) | Categorical | 125(12.72) | 121 | Prothrombin Time, sec, median(range) | Continuous | 12.0(11.4-12.9) |
| 49 | Bromhexine, n(%) | Categorical | 203(20.65) | 122 | International Normalized Ratio, median(range) | Continuous | 1.05(0.97-1.15) |
| 50 | Eucalyptol, n(%) | Categorical | 82(8.34) | 123 | White Blood Cell Count, ×10^9^/L, median(range) | Continuous | 5.70(4.11-7.95) |
| 51 | Irbesartan, n(%) | Categorical | 35(3.56) | 124 | Neutrophil Percentage, %, median(range) | Continuous | 75.3(63.9-84.6) |
| 52 | Metoprolol, n(%) | Categorical | 141(14.34) | 125 | Lymphocyte Percentage, %, median(range) | Continuous | 13.6(7.4-21.9) |
| 53 | Nifedipine, n(%) | Categorical | 125(12.72) | 126 | Monocyte Percentage, %, median(range) | Continuous | 7.75(4.90-11.17) |
| 54 | Amlodipine, n(%) | Categorical | 57(5.80) | 127 | Eosinophil Percentage, %, median(range) | Continuous | 0.20(0.00-1.00) |
| 55 | Furosemide, n(%) | Categorical | 131(13.33) | 128 | Basophil Percentage, %, median(range) | Continuous | 0.20(0.10-0.40) |
| 56 | Spironolactone, n(%) | Categorical | 53(5.39) | 129 | Neutrophil Count, ×10^9^/L, median(range) | Continuous | 4.30(2.74-6.35) |
| 57 | Sacubitril/Valsartan, n(%) | Categorical | 43(4.37) | 130 | Lymphocyte Count, ×10^9^/L, median(range) | Continuous | 0.78(0.50-1.18) |
| 58 | Atorvastatin, n(%) | Categorical | 118(12.00) | 131 | Monocyte Count, ×10^9^/L, median(range) | Continuous | 0.48(0.30-0.69) |
| 59 | Aspirin, n(%) | Categorical | 75(7.63) | 132 | Eosinophil Count, ×10^9^/L, median(range) | Continuous | 0.02(0.00-0.10) |
| 60 | Clopidogrel, n(%) | Categorical | 75(7.63) | 133 | Basophil Count, ×10^9^/L, median(range) | Continuous | 0.02(0.01-0.10) |
| 61 | Low molecular weight heparin, n(%) | Categorical | 339(34.49) | 134 | Red Blood Cell Count, ×10^12^/L, median(range) | Continuous | 3.98(3.51-4.40) |
| 62 | Enoxaparin, n(%) | Categorical | 85(8.65) | 135 | Hemoglobin, g/L, median(range) | Continuous | 121.0(104.0-133.0) |
| 63 | Nadroparin, n(%) | Categorical | 78(7.93) | 136 | Platelet Count, ×10^9^/L, median(range) | Continuous | 184.0(126.3-251.8) |
| 64 | Rivaroxaban, n(%) | Categorical | 40(4.07) | Infection Indicators | | | |
| 65 | Sulodexide, n(%) | Categorical | 78(7.93) | 137 | Infection Indicators | Categorical | 656(66.73) |
| 66 | Insulin, n(%) | Categorical | 147(14.95) | Output | | | |
| 67 | Metformin, n(%) | Categorical | 37(3.76) | 1 | Moderate to severe liver injury, n(%) | Categorical | 90(9.16) |
| 68 | Acarbose, n(%) | Categorical | 36(3.66) |  |  |  |  |

**Supplement Table 2. Patients’ characteristics in the RF and ET model for ML explainability**

| **Variables** | | **RF** | | **ET** | |
| --- | --- | --- | --- | --- | --- |
|  |  | **Figure 4A** | **Figure 4B** | **Figure 4C** | **Figure 4D** |
| Demographics | |  |  |  |  |
|  | Gender | Female | Male | Male | Male |
| Severity of COVID-19 at admission | |  |  |  |  |
|  | Severe | Yes | No | Yes | No |
| Treatment Duration | |  |  |  |  |
|  | Treatment duration, day | 12 | 9 | 15 | 12 |
| Medication | |  |  |  |  |
|  | Doxofylline | No | No | Yes | No |
|  | Low Molecular Weight Heparin | Yes | Yes | No | No |
|  | Diclofenac | No | No | No | No |
|  | Glycyrrhizin | Yes | No | No | Yes |
|  | Glutathione | No | No | No | No |
|  | Polyunsaturated Phosphatidylcholine | No | No | No | No |
| Laboratory results | |  |  |  |  |
|  | Alanine aminotransferase, U/L | 12 | 14 | 27 | 23 |
|  | Aspartate aminotransferase, U/L | 30 | 20 | 42 | 45 |
|  | Alkaline phosphatase, U/L | 80 | 73 | 63 | 97 |
|  | Gamma-glutamyl transferase, U/L | 38 | 42 | 78 | 126 |
|  | Total Protein, g/L | 59 | 60 | 64 | 45 |
|  | Albumin/Globulin Ratio | 1.25 | 1.66 | 0.84 | 1.97 |
|  | Total Bilirubin, μmol/L | 9.3 | 12.0 | 29.7 | 4.7 |
|  | Direct Bilirubin, μmol/L | 3.6 | 5.4 | 14.1 | 2.9 |
|  | Creatinine, U/L | 84 | 134 | 71 | 394 |
|  | Urea/Creatinine Ratio | 18.2 | 23.5 | 11.5 | 14.4 |
|  | Lactate Dehydrogenase, U/L | 978 | 511 | 346 | 300 |
|  | Neutrophil Percentage, % | 80.7 | 87.2 | 79.2 | 73.8 |
|  | Lymphocyte Percentage, % | 8.8 | 4.5 | 13.1 | 11.3 |
|  | Monocyte Percentage, % | 10.5 | 8.3 | 7.4 | 14.9 |
|  | Basophil Percentage, % | 0 | 0 | 0.2 | 0 |
|  | Neutrophil Count, ×10^9^/L | 3.69 | 7.35 | 7.5 | 1.4 |
|  | Eosinophil Count, ×10^9^/L | 0 | 0 | 0.01 | 0 |
|  | Red Blood Cell Count, ×10^12^/L | 3.67 | 4.54 | 5.52 | 3.35 |
|  | Hemoglobin, g/L | 107 | 146 | 159 | 108 |
| Outcome | |  |  |  |  |
|  | Moderate to severe liver injury | Yes | No | Yes | No |

**Supplement Table 3.** The 5-fold cross-validation diagnostic performance of the RF and ET model for online simplified version(mean±SD)

| **Model** | **AUC** | **Accuracy** | **Precision** | **Recall** | **F1-score** |
| --- | --- | --- | --- | --- | --- |
| Random Forest (RF) | 0.994±0.003 | 0.954±0.021 | 0.936±0.026 | 0.985±0.015 | 0.960±0.019 |
| Extra Trees(ET) | 0.992±0.004 | 0.961±0.016 | 0.956±0.027 | 0.975±0.021 | 0.965±0.014 |
